# Using a single penetrating interfascicular electrode to improve spatial selectivity of an extraneural polymeric cuff array

**DOI:** 10.1186/s42234-025-00193-6

**Published:** 2025-12-10

**Authors:** Imane Ben M’Rad, Zachary K. Bailey, Estelle A. Cuttaz, Rylie A. Green

**Affiliations:** 1 Bioengineering, Imperial College London, London, SW7 2AZ UK; 2 School of Life Sciences, Institute of Bioengineering, EPFL, Lausanne, Switzerland

**Keywords:** Stimulation selectivity, Nerve cuff electrode arrays, Conductive elastomers, Penetrating electrode, SPIFEC

## Abstract

**Background:**

Damage to the peripheral nervous system severely disrupts motor control, sensory perception, and organ function, often resulting in long-term disability. To restore such impairments, peripheral nerve interfaces (PNIs) aim to achieve precise stimulation selectivity, yet current approaches face several limitations. Most PNIs rely on metal-based electrodes, which introduce a mechanical mismatch with soft neural tissue and are limited by low charge-injection capacity. The device design of these PNIs also suffers from a fundamental trade-off: highly invasive approaches enable high selectivity but provoke strong foreign-body responses, while less invasive designs minimize tissue damage but fail to provide sufficient selectivity. Although current-steering strategies have been explored to enhance selectivity, their performance remains inadequate for clinical application. Significant advances in PNIs are required to safely achieve higher selectivity.

**Methods:**

In this work, a novel array consisting of a single penetrating interfascicular electrode (SPIF) added to an extraneural cuff (EC) array, termed SPIFEC, was developed using laser-based fabrication and polymeric materials. Electrochemical properties were characterized, and ex vivo experiments using whole rat sciatic nerve were conducted to assess fascicular selectivity. The implantation was assessed through computed tomography (CT) imaging.

**Results:**

The SPIFEC design includes seven extraneural electrodes and one double-sided interfascicular penetrating electrode. Electrochemical analysis revealed the polymeric electrodes had low impedance, high charge storage capacity and high charge-injection limit, when compared to previous reports on traditional metallic devices. Ex vivo studies demonstrated that the device achieved high fascicular selectivity, particularly in nerves with well-defined fascicles, outperforming a comparable non-penetrating cuff. CT imaging confirmed the interfascicular positioning of the penetrating electrode.

**Conclusion:**

These results demonstrate the potential of this novel SPIFEC array in enhancing spatial selectivity for peripheral nerve applications. Further studies, including chronic in vivo testing, are required to fully evaluate long-term performance and clinical potential in neuroprosthetic systems.

**Supplementary Information:**

The online version contains supplementary material available at 10.1186/s42234-025-00193-6.

## Background

**Fig. 1:**
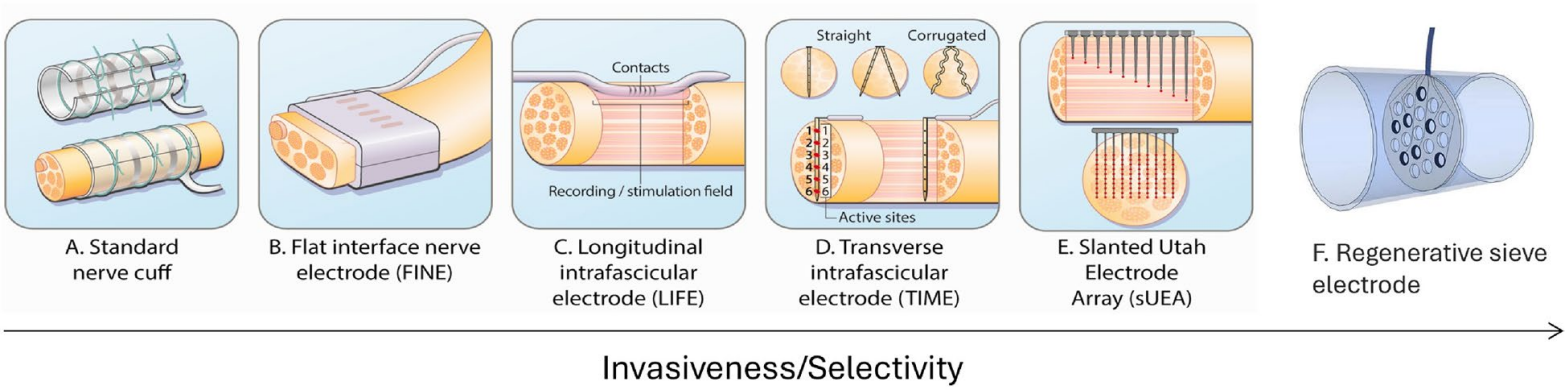
Type of PNIs with the according invasiveness and selectivity. **A**-**E** Adapted from Roche et al. (2023) licensed under CC BY-NC 4.0 and (**F**) Ortiz-Cataln et al. (2012), licensed under CC BY 2.0

Restoring function in the damaged or diseased peripheral nervous system is a key goal of bioelectronic medicine and neuroprosthetics. While electrical stimulation remains the most direct and widely used method for neural modulation, a critical challenge across most applications is achieving high spatial selectivity, that is, activating only the target nerve fascicles while minimizing off-target effects (Choi et al. 2001). A lack of selectivity can compromise both patient safety and therapeutic efficacy. For instance, the vagus nerve regulates a wide range of physiological systems, including respiratory, cardiac, endocrine, and immune functions (Yuan et al. 2016). While many neuromodulation devices target the vagus nerve (Yuan and Silberstein 2016; Courties et al., 2021), poor selectivity or suprathreshold stimulation parameters have been linked to serious adverse effects such as bradycardia and apnea (Fitchett et al. 2021). Selectivity is equally critical in the use of advanced prostheses, where precise sensory feedback is essential for improving device functionality and patient adoption. Among several contributing factors, low selectivity and particularly the absence of sensory feedback are major reasons why many amputees abandon prosthetic hands, with rejection rates reaching up to 40% (Stephens-Fripp et al. 2018). Selective stimulation of nerve fibers enables accurate transmission of tactile information from different parts of a prosthetic hand, enhancing functionality and dexterity while reducing the need for constant visual attention (Roche et al. 2023; Raspopovic et al. 2014). One way to enhance spatial selectivity relies on the design of the device interfacing with the peripheral nervous system. There exists a wide range of peripheral implants with different levels of invasiveness and selectivity, as shown in Fig. 1 (Roche et al. 2023). Nerve cuffs (Fig. 1A), or extraneural peripheral nerve interfaces (PNIs), are the least invasive way to interact with a nerve, encircling it externally without penetration. As they remain outside the epineurium without direct nerve access, their stimulation selectivity is limited.

Another design, the flat interface nerve electrode (FINE, Fig. 1B), reshapes the nerve in a rectangular shape to bring centrally located axons closer to the electrode surface for improved contact and selectivity (Tyler and Durand 2002). More invasive strategies have been investigated to target higher selectivity. For example, the slowly penetrating interfascicular nerve electrode (SPINE) implant was developed to insert electrode contacts within the epineurium of the nerve to have closer contact with the fascicles yet without penetrating the perineurium to avoid potential fascicle damage (Tyler and Durand 1997). Although more invasive peripheral nerve interfaces (PNIs) such as intrafascicular electrodes (e.g., LIFE, TIME, sUEA) offer better proximity to target axons and thus higher selectivity, their clinical adoption is limited by concerns over foreign body responses (FBR) and long-term stability (Yoshida et al. 2007; Boretius et al. 2010; Larson and Meng 2020; Branner et al. 2004). Interestingly, a recent study simulated the stress distribution around the nerve for both the standard TIME implant and a hybrid design, which combined a TIME implant in the center with an extraneural cuff-like structure surrounding the nerve. The simulations indicated that the hybrid design reduced peak stresses by 70% compared to the standard TIME implant. However, no further development or experimental testing of the hybrid device has been conducted (Akouissi et al. 2022). Finally, regenerative electrodes (Fig. 1F) are the most invasive type of peripheral nerve interface. They require transecting the nerve and rely on its intrinsic ability to regenerate through the electrode structure (2012). As the axons regrow through the microchannels or conduits, this configuration can potentially provide higher selectivity by establishing intimate contact with individual or small groups of fibers. Nonetheless, regenerative electrodes have not yet been evaluated in human clinical trials. While device design can enhance proximity to deeper neural structures, this improvement is inherently limited by the need to preserve nerve health, ultimately limiting fascicular and subfascicular selectivity.

An alternative or complementary approach uses current stimulation paradigms within the PNI to shape the electrical current distribution, enabling selective targeting of deeper nerve fibers. In cat experiments, Tarler et al. (Tarler and Mortimer 2004) used anodic/cathodic current steering stimulation with a four-contact nerve cuff to selectively recruit motor fascicles and were able to achieve more effective recruitment than single-contact stimulation. Similarly, Polasek et al. (Polasek et al. 2009) found that field steering with a multi-contact nerve cuff achieved greater selectivity in human muscle activation than single-contact stimulation. More recently, a soft multi-contact cuff with up to 16 electrodes showed promising results in in vivo studies with rat and pig sciatic nerves (Paggi et al. 2024). When using multiple electrodes, current steering approaches have been shown to better control the current spread and the volume of tissue activated. However, such devices remain insufficiently selective for many clinical applications, especially those being deployed on highly fascicularised nerves, such as the vagus.

A key consideration when designing implants for peripheral nerves is matching the inherent soft and flexible nature of nerve tissue. Peripheral nerves are subject to constant physiological motion, which means that implanted devices must be able to form a tight, conformal interface with a soft, dynamic environment. In addition to providing a stable interface, a proper fit enhances seal resistance at the device-nerve interface, preventing current leakage and signal contamination, critical for effective stimulation (González-González et al. 2018). Traditional PNI devices rely on stiff metallic electrode materials, which can result in poor contact with the nerve and reduced interface stability. The limited ability of metals to safely inject charge further impedes their stimulation efficiency (Zheng et al. 2021). One approach is the use of soft and flexible materials that more closely conform to nerve mechanics, enabling a more stable, well-sealed device-nerve interface. Recent research has focused on soft and flexible polymeric electrode materials such as conductive polymers (Shi et al. 2020) (Tang, et al., 2017). These materials offer excellent electrical properties, enabling high charge injection abilities for efficient stimulation without tissue damage. Their low stiffness decreases the mechanical mismatch between the device and the soft neural tissue, reducing the inflammatory response and improving stability and longevity of the implant (Green and Abidian 2015). A notable example is the conductive elastomer (CE), which combines the conductive polymer, PEDOT, with a softer, flexible elastomeric matrix (Cuttaz et al. 2019). Translation of this material into a functional tripolar cuff device using laser-based manufacturing processes has been developed by Cuttaz et al. (2021), and shown enhanced electrical functionality in vivo compared to traditional metallic cuffs. Using the same manufacturing methods, Bailey et al. (2025) developed an 8-electrode fully polymeric nerve cuff with electrodes circumferentially arranged around the nerve bundle. This design demonstrated promising preliminary fascicular spatial selectivity in rat sciatic nerves, highlighting the potential of polymer-based cuffs for stable and flexible interfacing. However, its extraneural configuration posed inherent limitations, and selectivity was limited as off-target activation could not be well constrained. The three limiting factors for selectivity were the purely extraneural electrodes that utilised only nearest neighbor stimulation, the relatively large size of the electrodes compared to the target fascicles, and the lack of a locking mechanism of the cuff around the nerve to minimize distance between electrode and target fascicle. These limitations resulted in activation of off-target fascicles before the entire target fascicle could be activated.

To address these shortcomings, this study sought to improve the 8-electrode cuff design by incorporating a small interfascicular penetrating electrode. It was hypothesized that coupling an extraneural cuff with a single, soft and flexible penetrating electrode would reduce the electrode-to-fascicle distance, thereby enhancing current steering resolution and improving fascicle-specific recruitment for all fascicle sizes. This hybrid approach aims to combine the stability and low invasiveness of extraneural cuffs with the improved selectivity of intraneural interfaces, potentially overcoming the trade-off between invasiveness and functional performance that limits existing peripheral nerve interfaces. Leveraging the CE material technology and existing laser fabrication techniques, SPIFEC was fabricated entirely of polymeric materials. This enabled not only conformal wrapping of the nerve, but also the ability to image the device in situ and understand the role of heterogeneous neuroanatomy on selective stimulation. Electrochemical characterization demonstrated superior functionality compared to literature reports on metallic devices, and ex vivo experiments showed promising fascicle selectivity.

## Methods

### Nerve cuff design and fabrication

The SPIFEC nerve cuff was fabricated based on previous work from (Cuttaz et al. 2021; Bailey et al. 2025). Briefly, a composite material of poly(3,4-ethylenedioxythiophene):polystyrene sulfonate (PEDOT:PSS) (Sigma) and polyurethane (PU) (Pellethane 2363–80AE Polyurethane Elastomer), with 25 wt% PEDOT:PSS loading was fabricated yielding a solvent cast sheet of conducting elastomer (CE) material. The nerve cuff was fabricated by first spin-coating (EMS 6000 Photoresist Spinner, Electronic Micro Systems) a sacrificial layer of poly(styrene sulfonic acid) (PSSA) (Sigma) on a glass slide. Then, a thin layer of polydimethylsiloxane (PDMS) (Sylgard 184, Dow Corning Corporation) was spin-coated on the glass slide. After curing, a thin CE sheet was manually applied on the partially cured PDMS layer. The electrode pattern of the nerve cuff was then laser-cut at 515 nm with a femtosecond micromachining laser system (Coherent Monaco, Optec Lasea Group). The excess CE was peeled off and a second layer of PDMS insulation was spin-coated and cured. The active and bonding sites were exposed by laser ablation of the insulating PDMS. The contour of the nerve cuff was laser-cut on the PDMS. Then, the glass slide was immersed in deionized water for device release and gently removed. Silver wires (125 μm diameter, PTFE insulated, Advent Research Materials) were bonded to the CE bonding pads and the contact zone between the silver wires and the bonding pads was insulated with silicone (MED4-4420, NuSil technology) and cured overnight at 40 °C. Finally, the silver wires were soldered to a pin socket connector that enabled electrical communication with a multichannel stimulator (STG 4008, Multi Channel Systems).

**Fig. 2:**
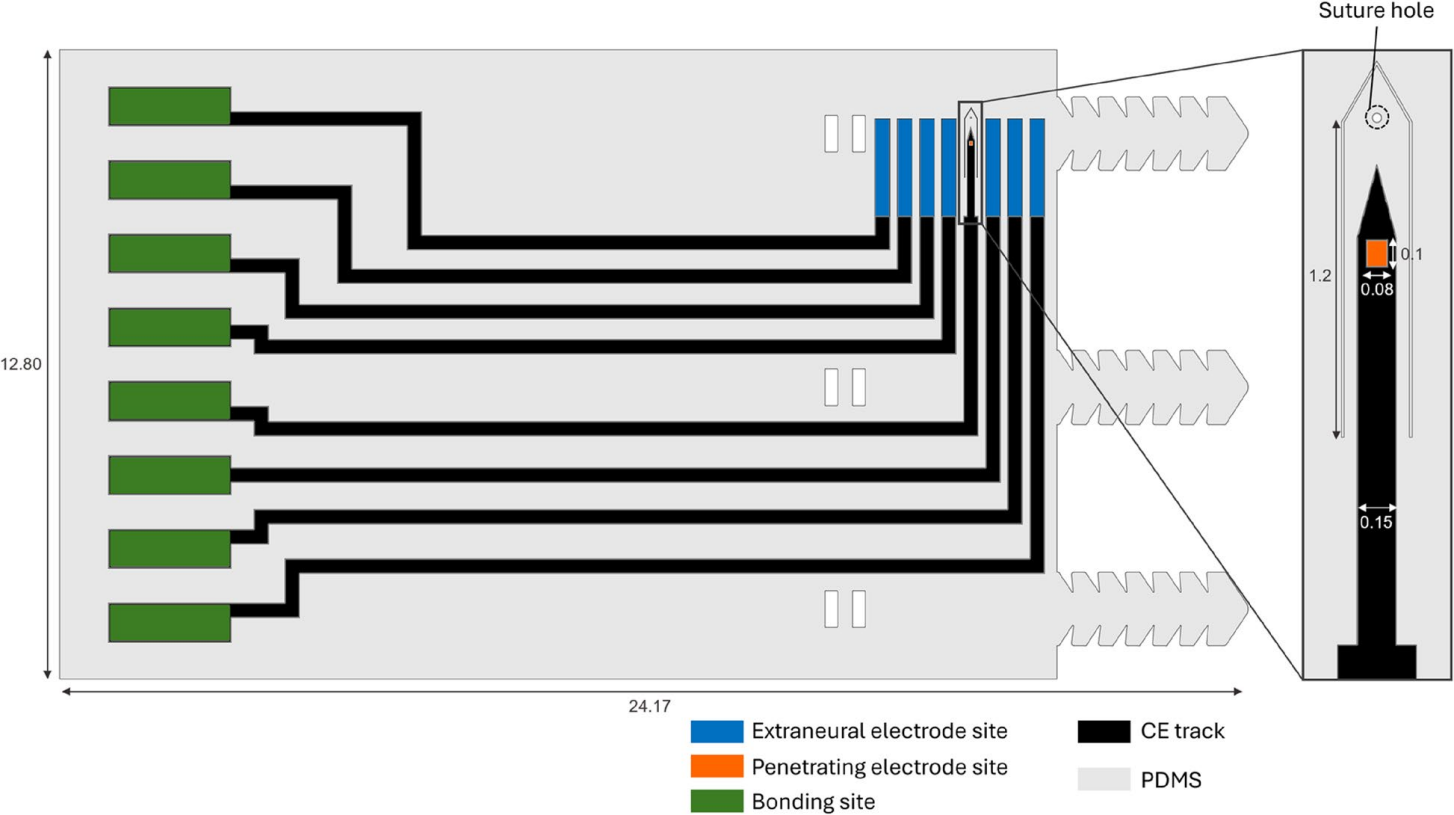
SPIFEC cuff design. Schematic of hydrid nerve cuff design with dimensions in mm. Electrode sites are shown in blue and orange while bonding sites are displayed in green. The light gray shade represents the PDMS contour around the cuff and the black tracks are CE. Details of the penetrating electrode are shown on the right rectangle of the image

Figure 2 illustrates SPIFEC’s design, consisting of seven extraneural electrodes (280 μm × 1980 μm) and a single double-sided interfascicular penetrating electrode (80 μm × 100 μm per side). To maintain continuity with the 8-electrode cuff design of Bailey et al. (2025), one extraneural site was replaced with the penetrating electrode. The latter was fabricated with tapered CE and PDMS tracks to facilitate smooth insertion into the nerve while minimizing the risk of breakage or delamination. Its PDMS tip incorporated a suture hole, enabling traction-assisted insertion. For secure implantation, a belt-loop ratchet system was integrated, allowing sutureless closure of the cuff around the nerve.

The nerve cuff was electrochemically characterized by performing electrochemical impedance spectroscopy (EIS) and cyclic voltammetry (CV) using a potentiostat (Multi Autolab/M101, Metrohm). A three-electrode setup was used with a Ag/AgCl reference electrode (ET072, eDAQ), a platinum rod counter electrode (1 cm diameter rod, 6 cm length) and the electrode active site of the device as the working electrode, all immersed in phosphate-buffered saline (PBS) as the electrolyte. EIS measurements were performed with sinusoidal excitation voltage of 30 mV at frequencies ranging from 1 to 10^5^ Hz. CV was conducted at a scan rate of 0.15 V/s for a total of 5 scans and a potential range of −0.6 to 0.8 V.

### Ex vivo characterization

The ex vivo study was based on Bailey et al. (2025). Female Sprague–Dawley rats (weighing 200–230 g, Charles River, UK) were humanely euthanized under isoflurane anesthesia. Female rats were used in alignment with an ongoing study within the research group to minimize additional animal use and reduce overall animal consumption. Both sciatic nerves were harvested from their base near the spinal cord distally to the end of their three largest fascicles, namely the tibial nerve (TN), the peroneal nerve (PN), and the sural nerve (SN). In total, five trials were carried out with five different nerves. The nerve was then placed in oxygenated modified Krebs–Henseleit buffer, cleaned from fat and excessive tissues and its ends were sutured. Each fascicle was identified by its anatomical difference, being the general size. The sural fascicle was expected to be the thinnest, the tibial the longest and thickest and the peroneal intermediate in size. Additionally, the peroneal branches and divides more proximally compared to the other fascicles resulting in the shortest segment after explantation.

An epineurial window was cut to facilitate the insertion of the penetrating electrode. The nerve cuff was prepared beforehand by using the suture hole to attach the tip of the penetrating electrode to a 9–0 suture (V100-3 needle, W2898, Ethicon) by tying a double knot. Using a microscope (SMZ 745, Nikon), the needle was inserted between the tibial and peroneal fascicles, and the suture was pulled until the penetrating electrode passed through the nerve and reached its center. Precisely, the penetrating electrode was slowly pulled inside the nerve until the double knot was observed on the other side of the nerve. The nerve cuff was then wrapped around the nerve and its fit was secured by closing the belt loop and tightening the ratchets to achieve full closure and end sealing.

**Fig. 3:**
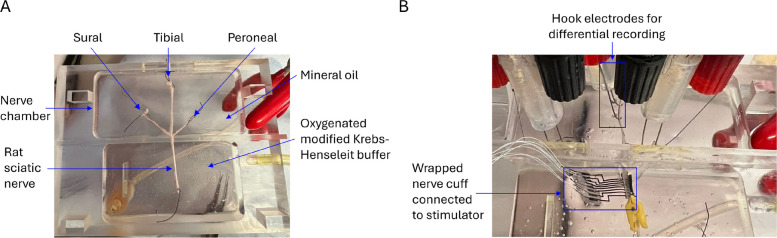
Experimental setup of the ex vivo study. **A** Rat sciatic nerve placed in the nerve chamber in oxygenated modified Krens-Henseleit buffer. Fascicles are placed in the recording side in mineral oil. **B** SPIFEC wrapped on nerve with implanted penetrating electrode. Device is connected to the stimulator. Hook electrodes are placed on each fascicle for differential recording

Figure 3 illustrates the experimental setup for the ex vivo study. The nerve was placed in the custom-designed nerve chamber, consisting of two cavities separated by a wall with a feedthrough hole of 1.2 mm. The first chamber contained the sciatic nerve while the second chamber contained the 3 fascicles, fixed in position with insect pins. The first chamber was filled with continuously reoxygenated modified Krebs–Henseleit Buffer using a peristaltic pump (Pump P-1, Pharmacia Biotech). The second chamber was filled with mineral oil and a pair of Ag/AgCl-coated custom-built recording hook electrodes placed on each fascicle for recording of compound nerve action potentials (CNAPs). The distance between each pair of hooks was set to 5 mm and remained constant across all trials. A small metallic pin was fixed in the nerve chamber and placed contacting the nerve epineurium between the stimulation and the recording sites, then connected to the ground of the stimulator to minimize the recording of stimulation artifact (Panskus et al. 2023). Silicone grease was used on the feedthrough hole to prevent liquid leakage from one side of the chamber to the other.

The nerve cuff was connected to the stimulator. Each hook electrode pair was linked to a differential gain amplifier (DP-304A, Warner Instruments) and 50–60 Hz noise was filtered using a Humbug device (Digitimer). Signals were digitized through a data acquisition system (Micro3 1401, Cambridge Electronic Design Ltd.) and analyzed with Spike2 software. Eight balanced biphasic stimulation protocols were applied, varying injection and return pulse widths from 50 to 700 μs and employing both bipolar and tripolar configurations with either anodic-first or cathodic-first stimulation on the penetrating electrode. The penetrating electrode remained active throughout, while external electrodes were sequentially activated across protocols. Stimulation amplitudes ranged from 0 to 1600 μA in 50 μA steps, delivered in randomized order at 25 Hz. Each stimulation condition was repeated five times. Supplementary details can be found in Table S1 in the Appendix.

Evaluation of the charge-injection limit (CIL) of the device on the nerve was conducted by delivering charge-balanced cathodic-first pulses with varying pulse widths (100–1000 µs) using the stimulator (STG4002-16 mA, Multichannel Systems). The CIL was determined as the quantity of charge required to polarize the electrode–electrolyte interface to the water reduction potential (−0.6 V) by progressively increasing the stimulation amplitude until reaching the potential threshold.

### Micro-CT scan imaging

Upon completion of the ex vivo stimulation protocols, the position of the SPIFEC device around the nerve was preserved by casting alginate directly within the nerve chamber. Specifically, 8 g of alginate (Vesey Gallery) was mixed with 32 mL of Lugol’s solution (Sigma) and poured over the sciatic side of the nerve chamber. Lugol’s solution was used to enable contrast enhancement for subsequent computed tomography (CT) imaging. Once cured, the alginate block was removed and immersed in Lugol’s solution for an additional 36 h of staining. The alginate block was wrapped in cling film to prevent dehydration and placed in a micro-CT scanner (Zeiss Xradia Versa 510) for imaging. Scans were acquired using 0.4 × magnification, with a voltage of 80 kV, power of 7 W and an exposure time of 6 s.

### Data analysis

Data processing followed the same procedure as described in previous experiments (Bailey et al. 2025). For each segment, signals were averaged across five repetitions and filtered to remove the stimulation artifact. CNAPs were identified using a peak detection algorithm (Matlab), with peak-to-peak voltage (V_pp_) defined as the difference between the signal's maximum and minimum. Each CNAP was normalized to the maximum response within its fascicle, yielding a normalized CNAP (V̂_pp_). Fascicular selectivity was then calculated using Eq. 1, where the normalized response of the target fascicle was reduced by the average response of off-target fascicles:

1 $${Sel}_{i}={\widehat{V}}_{pp,target } - \frac{1}{2} \sum_{j\ne i}{\widehat{V}}_{pp,off,j}$$

where in index *i* indicates the target fascicle, and *j* represents off-target fascicles. For each stimulation protocol, the highest selectivity was retrieved per fascicle. Three-way ANOVA followed by post hoc Tukey tests, as well as Student’s t-tests, were performed to compare selectivities. Statistical significance was set at *p* < 0.05. The Shapiro–Wilk test was used to verify that the residuals of the ANOVA models were approximately normally distributed (*p* > 0.05), supporting the use of parametric tests.

## Results

### Nerve cuff design and fabrication

**Fig. 4:**
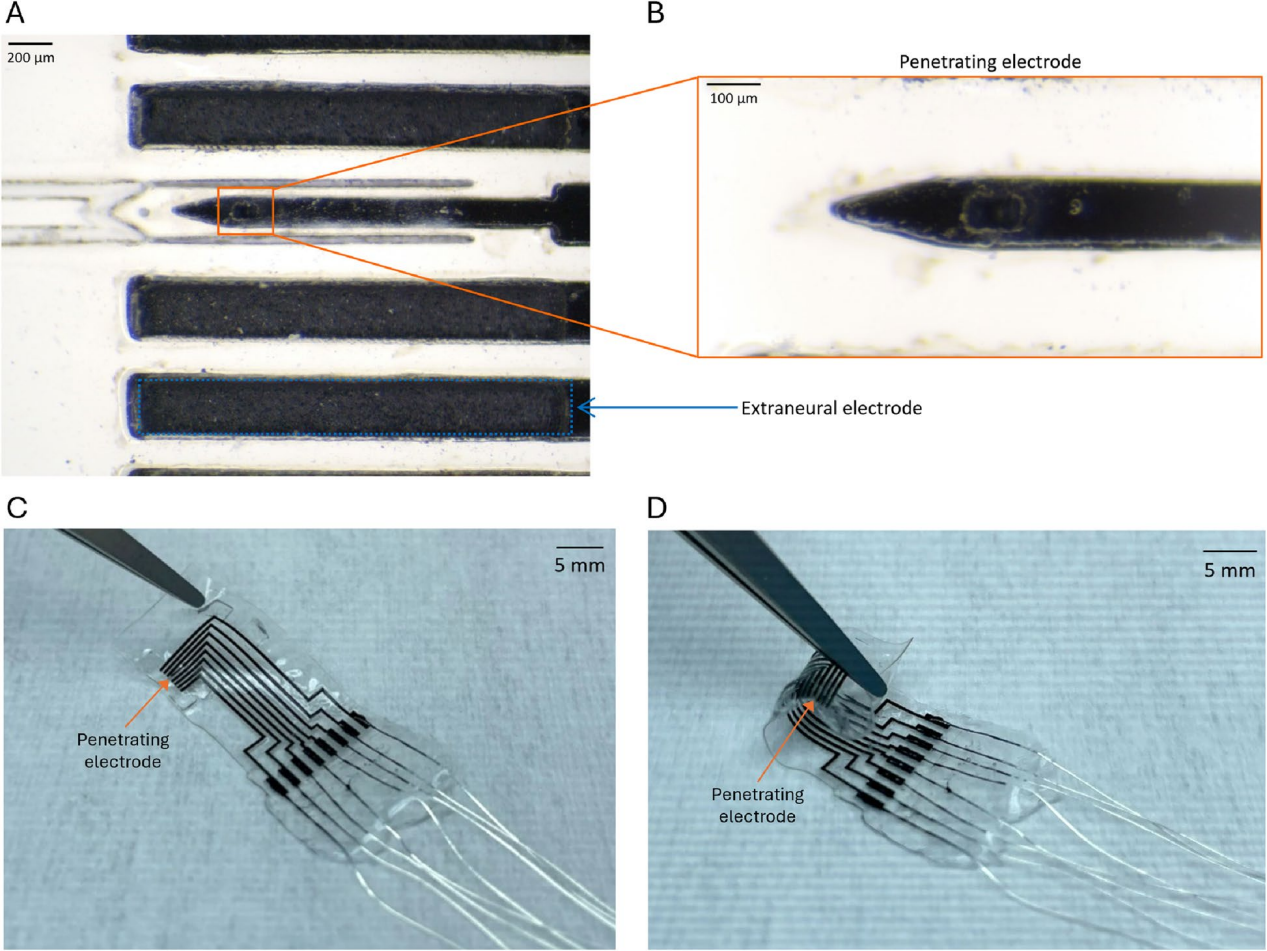
SPIFEC fabrication (**A**) Images of the external electrode (blue rectangle) and penetrating electrode (orange rectangle) are shown. **B** Zoomed image of penetrating electrode. Images of (**C**) the complete hybdrid penetrating nerve cuff SPIFEC, and (**D**) once wrapped. Note that the ratchets were not present on (**C**) and (**D**). Orange arrows indicate the location of the penetrating electrode

SPIFEC was designed to have one double-sided SPIF of 0.008 mm^2^ for each side for a total exposed area of 0.016 mm^2^ and 7 extraneural electrodes, with a surface 0.55 mm^2^ each (Fig. 4A-B). The device’s flexibility facilitated its wrapping around a nerve (Fig. 4C-D). The total average thickness of the device (including conductive and insulative layers) was 221.0 ± 24.0 μm.

### Electrochemical characterization

**Fig. 5:**
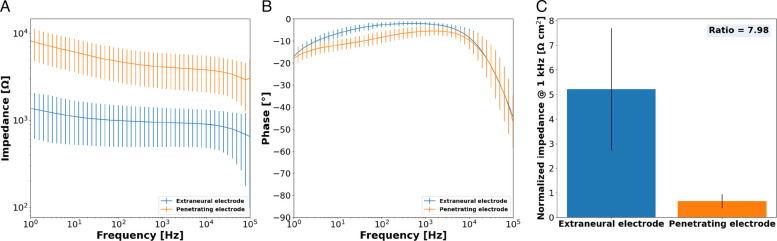
Electrochemical impedance spectroscopy performance showing (**A**) the impedance magnitude of the external (*n* = 98) and penetrating site (*n* = 15), (**B**) impedance phase for external and penetrating active site and (**C**) normalized impedance magnitude at 1 kHz of the external and penetrating site with regard to their respective surface area. Error bars denote one standard deviation

EIS was performed to determine the impedance magnitude and phase of both types of electrode designs i.e., external and penetrating electrodes. Both electrode types had similar impedance profiles across varying frequencies but displayed different magnitudes (Fig. 5A), with the impedance of the external electrode lower than the SPIF. At 1 kHz, the external site had an absolute impedance of 940.68 ± 446.11 Ω, whereas the penetrating site showed a 3.31-fold increase in impedance measured at 4086.53 ± 1781.36 Ω. Both phase plots looked similar in shape, with the phase values close to 0 degrees with decreasing frequencies (Fig. 5B), which is typical from conductive polymer-based materials. Impedances were normalized to their respective geometrical surface area (GSA) for a better comparison. The external sites yielded an average impedance of 5.22 ± 2.47 Ω cm^2^ at 1 kHz while the SPIF displayed an average impedance of 0.65 ± 0.29 Ω cm^2^ across multiple devices (Fig. 5C). The ratio between both electrodes was 7.98 irrespective of their GSA. For comparison, a previous study reported that platinum electrodes in a nerve cuff exhibited a normalized impedance of 27 Ω cm^2^ at 1 kHz (Cuttaz et al. 2021).

**Fig. 6:**
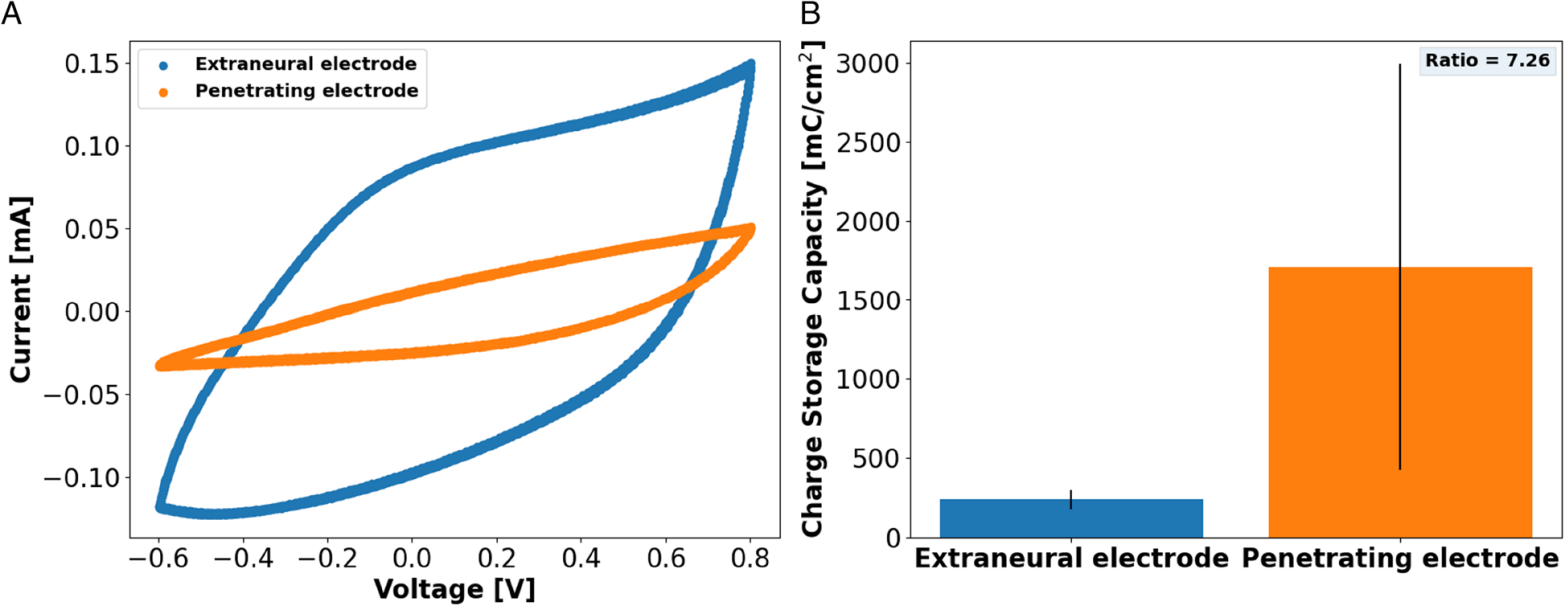
Cyclic voltammetry of the external and penetrating electrodes showing (**A**) the cyclic voltammograms of both electrode types and (**B**) the charge-storage capacity of the external (*n* = 98) and the penetrating electrode (*n* = 15)

CV was conducted and CSC of the SPIF and extraneural electrodes (Fig. 6A) were computed from the CV waveforms. The mean CSC was significantly higher for the SPIF (1707.77 ± 1284.38 mC/cm^2^) compared to the extraneural active site (235.29 ± 61.97 mC/cm^2^), with the SPIF exhibiting a 7.26-fold increase (Fig. 6B). Smooth platinum and platinum iridium microelectrodes typically have CSC in the 0.55 to 4 mC/cm^2^ range (Green et al. 2019; 2012).

### Ex vivo characterization

Ex vivo experiments were performed on rodent sciatic nerves where the sciatic bundle was stimulated across a wide range of parameters with the SPIFEC array. Neural signals were recorded from its three largest fascicles, namely the tibial, peroneal, and sural nerves. Activation percentage of the fascicles and their selectivity were then computed (Eq. 1 in section Data analysis) to assess the selectivity of a penetrating cuff design as well as the configuration and stimulation parameters yielding the highest selectivity. The configuration of the nerve cuff within the nerve was then imaged using micro-CT scan.

**Fig. 7:**
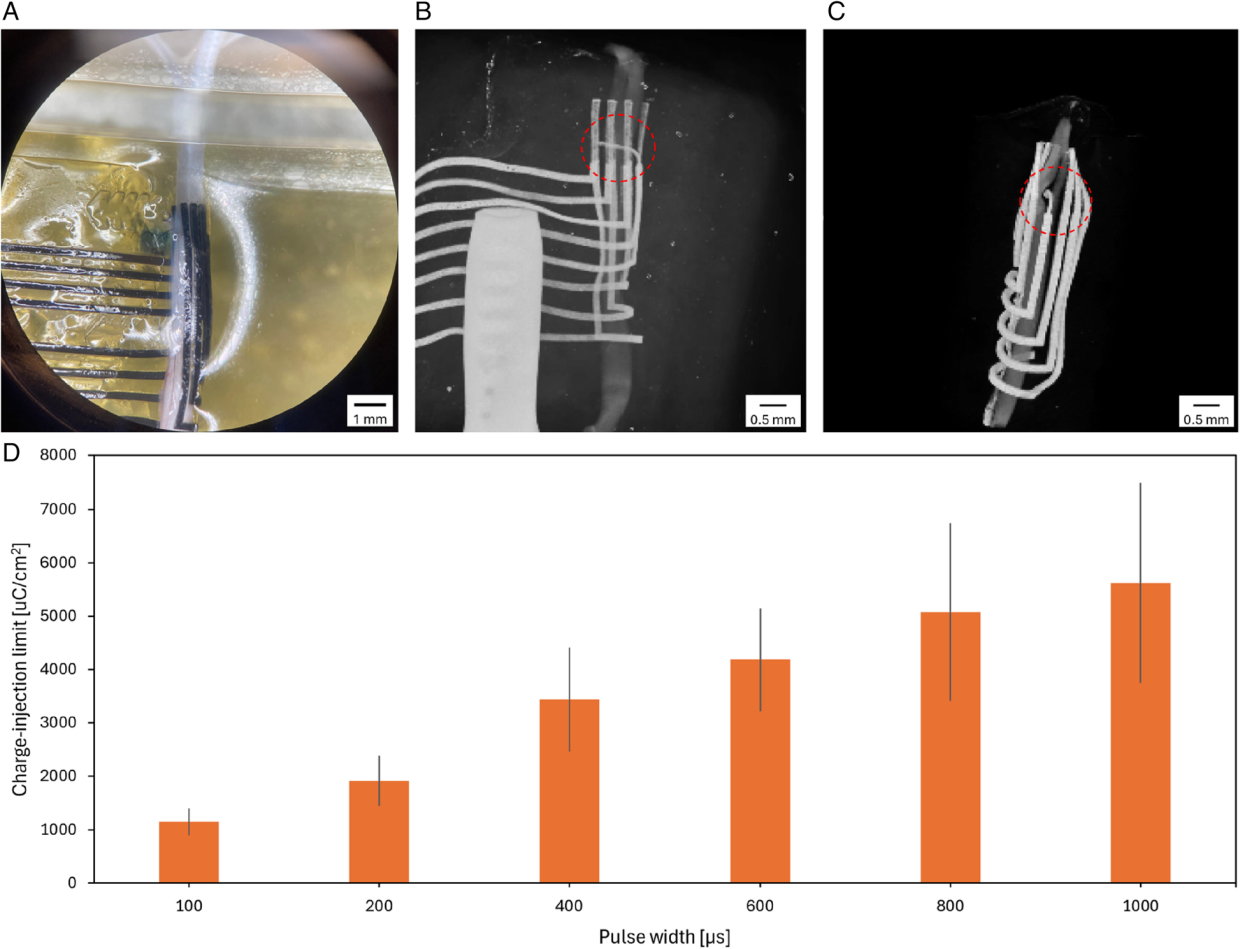
Ex vivo placement of SPIFEC onto rat sciatic nerve. **A** Microscope picture of the penetrating nerve cuff implanted onto the nerve. **B** Micro-CT imaging showing the PE track bent almost perpendicularly into the nerve (circled in red). The external electrodes are parallel to the nerve epineurium. Note that a plastic clamp (bottom left corner) was used in this trial to secure the cuff wrap around the nerve. **C** 3D reconstruction of the nerve cuff and the penetrating electrode within the nerve. The electrode tracks are in white and the nerve in dark grey. The penetrating electrode is inserted interfascicularly (circled in red). **D** CIL measurements of the penetrating electrode in ex vivo conditions with a stimulation pulse width range from 100 to 1000 μs (*n* = 3). Error bars represent mean ± 1 SD

The nerve implanted with the penetrating nerve cuff was imaged with a microscope (Fig. 7A) and micro-CT (Fig. 7B). Three-dimensional reconstruction revealed that the SPIF on the nerve cuff was stably inserted interfascicularly into the nerve (Fig. 7C). Recordings from the 3 fascicles were obtained, confirming that no major damage was caused to the fascicles during the SPIF insertion. CIL values were measured using voltage transient analysis to assess the stimulation efficacy of SPIF once inserted into the nerve. Biphasic stimulation with a pulse width of 100 μs resulted in a CIL of 1145.83 ± 252.59 μC/cm^2^, which increased to 5625.00 ± 1875.00 μC/cm^2^ at a pulse width of 1000 μs (Fig. 7D), confirming SPIF stimulation functionality.

**Fig. 8:**
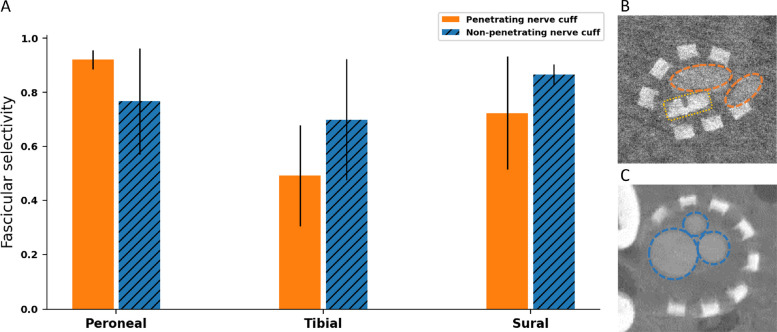
Ex vivo fascicular spatial selectivity performance for the best stimulation configurations, comparing the penetrating and non-penetrating nerve cuffs (**A**) Highest fascicular selectivity across stimulations of the penetrating and non-penetrating nerve cuff. Bars represent mean ± 1 SD; *n* = 5 per group. **B** Representative micro-CT cross-section of the rat sciatic nerve with the electrodes of the penetrating nerve cuff. Penetrating electrode is shown in yellow rectangle. Peroneal and tibial fascicle are circled in orange dashed ellipses. **C** Representative micro-CT cross-section of the rat sciatic nerve with the electrodes of the non-penetrating nerve cuff around it. Peroneal, tibial and sural fascicles are circled in blue dashed ellipses

Figure 8 illustrates the ex vivo fascicular spatial selectivity of SPIFEC assessed across 5 nerve trials. The selectivity for each fascicle was averaged across nerve trials and the best stimulation configuration per fascicle resulted in selectivity values of 0.92 ± 0.04 for the peroneal fascicle (asymmetric cathodic-first pulse bipolar configuration with pulse width variation on the injection contact; Stimulation 5, Table S1), 0.49 ± 0.19 for the tibial fascicle (symmetric cathodic-first bipolar configuration with pulse width variation on both injection and return contacts; Stimulation 1, Table S1), and 0.72 ± 0.21 for the sural fascicle (asymmetric cathodic-first bipolar configuration with pulse width variation on the return contact; Stimulation 2, Table S1) (Fig. 8A). These results were compared to a previous study that used a similar nerve cuff design that included eight external stimulation electrodes but without the SPIF, referred as the non-penetrating nerve cuff (Bailey 2025). The fascicular selectivities were similarly calculated and yielded 0.77 ± 0.20, 0.70 ± 0.22 and 0.87 ± 0.04, respectively for the peroneal, tibial, and sural fascicle for the best stimulation configuration per fascicle. While the mean value of peroneal selectivity was higher in the penetrating cuff compared to the non-penetrating cuff and lower for the tibial and sural fascicles, the differences were not statistically significant as p-values were respectively 0.15, 0.17 and 0.17 for the three fascicles. Cross-sections of nerves, each implanted with one of the two device types, were visualized using micro-CT for comparison. At the site of the SPIF insertion, two fascicles are observed, namely the tibial and peroneal (Fig. 8B), while all three fascicles are visible at the implantation site of the non-penetrating nerve cuff (Fig. 8C). The presence of only two fascicles at the penetrating electrode site is due to the choice of the implantation location. At this location, full fascicularisation has not occurred yet and the third fascicle emerges more distally along the nerve.

**Fig. 9:**
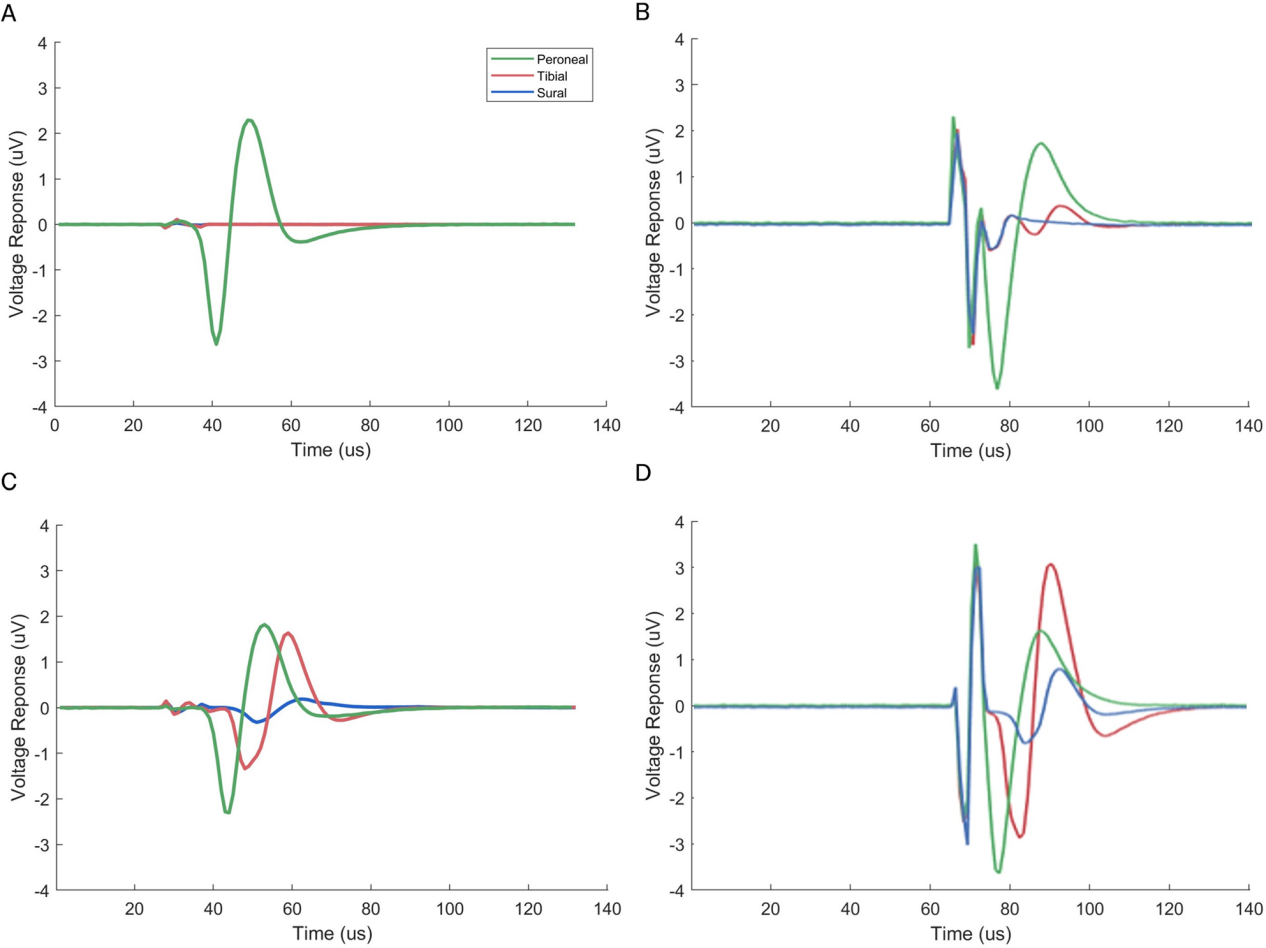
Representative compound nerve action potentials (CNAPs) recorded from the peroneal (green), tibial (red), and sural (blue) fascicles during selective and non-selective stimulation using penetrating and non-penetrating nerve cuffs. **A** Selective stimulation of peroneal fascicle with penetrating cuff. **B** Selective stimulation of peroneal fascicle with non-penetrating cuff. **C** Non-selective stimulation of peroneal fascicle with penetrating cuff. **D** Non-selective stimulation of peroneal fascicle with non-penetrating cuff. The first sharp peaks correspond to the stimulation artifact. The smooth peaks are the neural responses

Representative CNAP recordings for selective (Fig. 9 A and B) and non-selective stimulation (Fig. 9 C and D) obtained with the penetrating and non-penetrating cuffs are shown in Fig. 9. The stimulation artifacts were markedly reduced when using the penetrating cuff, owing to the use of a metallic pin that improved signal quality and facilitated data analysis and peak detection.

**Fig. 10:**
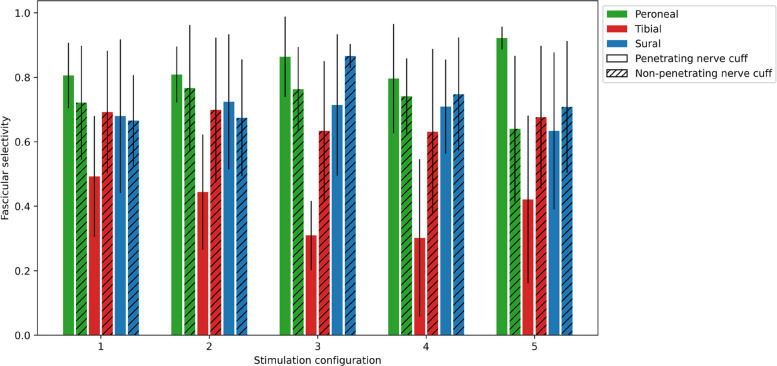
Comparison of ex vivo fascicular spatial selectivity performance between cuff types across all stimulation configurations. Bars represent mean ± 1 SD; *n* = 5 per group

Considering all stimulation configurations, the highest selectivities of both cuffs were compared (Fig. 10). While no statistically significant differences were found in peroneal selectivity between cuffs, the penetrating cuff yielded higher mean values across all stimulation configurations. In contrast, tibial selectivity when averaged across all configurations was significantly greater with the non-penetrating cuff. Sural selectivity values were comparable between both designs, though the best cuff configuration varied depending on the stimulation configuration. The corresponding ANOVA results are reported in Tables S2-S5 in the Appendix. It is noted that although the two cuff types can be compared, the SPIFEC cuff would enable the functionality of both, as it is possible to use the extraneural electrodes without the SPIF when required. Stimulation details can be found in Table S1 in the Appendix.

## Discussion

Improving spatial selectivity when interfacing with the peripheral nervous system is crucial for achieving finer neuromodulation in bioelectronics. This work presents the design and manufacturing of a novel cuff with a penetrating electrode called SPIFEC. A rationale design was envisioned based on the need for conformal nerve cuffs with improved discreet activation across peripheral nerve architectures. SPIFEC was laser fabricated, characterized and functionally evaluated in an ex vivo whole nerve preparation. The device was found to be functional with a low impedance, high charge-storage capacity, and high charge-injection limit. The nerve cuff design and insertion mechanism enabled a safe and stable interfascicular insertion into the nerve without causing major damage to the fascicles evidenced through the ex vivo nerve response and CT-scan images. SPIFEC, combining external and penetrating electrodes, showed promising fascicular selectivities when applied to an ex vivo whole sciatic nerve.

SPIFEC included two types of electrodes: a penetrating electrode designed to penetrate the nerve and interface directly with the fascicles, and extraneural electrodes configured to wrap around the nerve. The aim of this design was to better shape the electrical field to reach higher selectivity. Electrochemical characterization of the electrodes confirmed their functionality post-manufacturing. Both extraneural and penetrating electrodes demonstrated lower impedance and higher charge storage capacity (CSC) compared to previously reported values for platinum electrodes in the literature. Although the geometric surface area (GSA) of the extraneural electrodes was 34 times larger than that of the penetrating electrode, they only exhibited a reduced absolute impedance by a factor of 3.3, indicating a nonlinear relationship. Larger electrode surface area is generally correlated with improved electrochemical performance due to the increased available sites for charge transfer. However, when normalized for surface area, the smaller penetrating electrode demonstrated superior performance, with mean impedance magnitude and CSC differing by factors of 7.98 and 7.26, respectively. This suggests that electrochemical performance is not solely determined by geometric surface area. Previous studies have shown that smaller electrodes often exhibit higher CSC, partly due to edge effects (Cogan 2008; Green et al 2012; Ganji et al. 2017). Charge accumulates at the edge of the electrode and generates a non-uniform and concentrated electric field which leads to more efficient charge accumulation and delivery. As a consequence, this effect can allow sensory percepts to be elicited with less charge.

Another key parameter is the laser exposure of the active sites. During this step, while the aim is to remove the upper insulative layer, partial ablation of the underlying conductive elastomer usually occurs. This laser ablation likely roughens the surface of the polymer, further increasing the electrochemical surface area (ESA) of the electrode i.e. the effective surface area available for charge transfer is greater. However, the laser ablation profile is influenced by the size of the feature being processed, with the SPIF having substantially smaller dimensions compared to the external electrodes. Therefore, different laser parameters, including power, frequency, and marking speed, were required to efficiently expose the active sites. This size-dependent laser ablation is due to scaling effects in thermal diffusion and energy density distribution, which will impact heat dissipation and surface morphology of the surface being ablated. It is likely that the smaller electrodes obtained a more roughened ablation profile, further enhancing their electrochemical performance. Overall, the combination of micrometer-scale dimensions and laser ablation likely explains the superior performance of the SPIF compared to the extraneural electrodes. Scanning electron microscopy (SEM) or profilometry could be used to better quantify the true ESA, and enable a more accurate normalization of charge impedance values.

The device was implanted ex vivo in sciatic nerves to evaluate the stimulation selectivity enabled by SPIFEC. Upon device insertion into the excised nerve, CIL measurements were conducted to confirm the stimulation capability of the penetrating electrode. The ex vivo CILs of the penetrating electrode ranged from 1145.83 μC/cm^2^ to 5625.00 μC/cm^2^, depending on the pulse width. These values are notably high compared to conventional materials such as platinum and its alloys, which typically exhibit CILs between 50 and 150 μC/cm^2^ for a 200 μs pulse width stimulation (Cogan 2008). Surface modification and coatings techniques involving iridium oxide and PEDOT have been employed to improve CILs of metals and achieved values of 5000 and 15,000 μC/cm^2^, respectively (Won et al. 2018). However, such coatings carry a risk of delamination, which can degrade device performance, reduce long-term stability, and potentially induce tissue damage (Cogan 2008; Yang et al. 2021). In contrast, the proposed nerve cuff made from homogenous sheets of PEDOT-based conductive elastomer reaches a comparable range of CIL values while using a minimal electrode footprint and without relying on coating. This reduces the risk of mechanical failure, enhances device durability, and enables higher stimulation currents without compromising tissue safety.

Fascicular responses were recorded to assess the significance of the SPIF for selectivity. The results of SPIFEC were compared to those of a similarly fabricated cuff where all electrodes were extraneural and none penetrated the epineurium. Comparing this extraneural, non-penetrating cuff to SPIFEC aimed to assess whether the internal current steering yielded higher selectivity. When choosing the best stimulation configuration for each cuff and fascicle, the results showed a higher though not statistically significant peroneal selectivity with SPIFEC with a very low standard deviation across nerve trials. The non-penetrating cuff exhibited a statistically non-significant increase in selectivity for the tibial and sural fascicles. One possible explanation for the latter is the device implantation location. Nerve cross-section imaging revealed that all three major fascicles were visible with the non-penetrating cuff, whereas only the tibial and peroneal fascicles were observed at the site of the SPIF in SPIFEC, suggesting incomplete fascicularisation of the tibial section, with the sural fascicle still embedded within it. This anatomical difference was likely a confounding factor in the spatial selectivity performance of SPIFEC, particularly for the tibial fascicle, as it is the largest within the sciatic nerve. Since the sural portion has a smaller perineurium thickness and cross-sectional area, prior studies have shown it requires less current for selective activation (Davis et al. 2023). Therefore, higher sural selectivity was reached compared to the tibial fascicle. In contrast, the larger tibial fascicle requires higher current, likely resulting in co-activation of the sural region within it.

Future work will assess co-activation of fascicles to better understand this hypothesis. When comparing all stimulation protocols between cuff types, the higher peroneal selectivity for the penetrating nerve cuff is maintained across all stimulation protocols. It indicates that a well fascicularised nerve with separated fascicles such as the peroneal in this case stimulated by a penetrating nerve cuff reaches higher selectivity with minimal variability across animals compared to a similar cuff without a penetrating element. Additional experiments involving a larger number of nerves are needed, since the limited small sample size in the present study prevented statistical significance from being reached. Moreover, subsequent studies will quantify the maximum activation percentage achievable for each target fascicle with SPIFEC, while varying the allowable activation levels of off-target fascicles. Indeed, depending on the application and implantation site, different thresholds for acceptable off-target activation may be perceived. To enable a more accurate comparison of the selectivity achieved by each device, ex vivo experiments should be conducted with SPIFEC placed more distally, where the nerve is more fascicularised and the sural fascicle is separated from the tibial.

To enable such experiments, it is necessary to be able to accurately place SPIFEC and control device location during implantation. A more controlled insertion mechanism for the SPIF must be investigated to visualize the fascicularisation before implantation, potentially with techniques such as optical coherence tomography (Carolus et al. 2021; 2020). The information obtained from the micro-CT scans confirmed the interfascicular insertion of the device (without penetrating the actual neural fascicle) and this is likely to be less damaging than an intrafascicular device such as the TIME or LIFE implant. For instance, implantation of a Pt–Ir LIFE electrode was associated with a 40% reduction in axon diameter and a decrease in myelination at the implant site (Jung et al. 2018). Similarly, implantation of a TIME device led to a reduction in the number of myelinated fibers (Badia et al. 2011). The generation of CNAPs by the fascicles during the ex vivo experiments indicates that the SPIF insertion did not cause significant damage. To better assess insertion-related trauma and foreign body response, acute and chronic in vivo studies should be investigated.

Another important consideration of SPIFEC is that the penetrating electrode integrated into the nerve provides an anchoring point that helps maintain the cuff position relative to the nerve and its fascicles, conferring stability to the device-nerve interface. Previous studies on peripheral nerve interfaces have reported rotational displacement of nerve cuffs over time (Goodall et al. 1991), which can alter the alignment between electrode contacts and nerve fibers. Such displacement compromises the consistency of stimulation and may require repeated recalibration, which could be frustrating for the user and limit long-term functionality. In vivo experiments would also allow the evaluation of device stability upon limb movement. Additionally, further mechanical characterization, including uniaxial and fatigue tensile testing, is also critical to evaluate the structural performance of the device, especially the design of the penetrating electrode.

Future nerve cuff designs could include multiple penetrating electrodes shanks or a shank with multi-depth electrodes in attempt to better steer current and increase spatial selectivity. The laser used for the device fabrication provides a resolution of approximately 15 μm for conductive tracks and 30 μm for active sites. As this device was developed as a proof-of-concept, each side of the penetrating shank integrated only one active site for simplicity. Incorporating multiple active sites on the same shank would be a valuable improvement for future iterations. With the current laser resolution, it is feasible to pattern multiple active sites connected to a common track. However, implementing independently addressable active sites, each connected to a separate track and bonding pad, would require modifications to the current fabrication process.

Improving spatial selectivity is a key step forward for bioelectronics, as it reduces off-target effects and enhances stimulation efficiency. This is especially important in neuroprosthetic applications, where precise control over nerve activation can enable more natural motor functions and restore tactile feedback, potentially lowering cognitive load and reducing dependence on visual cues. The proof-of-concept polymeric SPIFEC device demonstrated in this study exhibited significant electrochemical performance, effective stimulation capabilities, and promising ex vivo selectivity. Increased selectivity may support safer long-term use by limiting unintended stimulation of surrounding tissues and associated side effects, paving the way for more refined and patient-specific neurorehabilitation therapies.

## Conclusion

In conclusion, SPIFEC is the first proof-of-concept nerve cuff integrating a single penetrating interfascicular electrode within an extraneural cuff, fully fabricated from polymer-based conductive and insulating materials. The design using laser-based fabrication techniques demonstrated significant electrochemical performance with low impedance and high charge-storage capacity and achieved high spatial selectivity in ex vivo rodent trials for well-fascicularised nerves, showing the potential for better performance compared to the control non-penetrating nerve cuff. Future studies will include larger sample sizes and evaluate performance with changes to the electrode design to ensure the penetrating elecrode is within the fascicularised space. The next step involves in vivo studies to evaluate the foreign body response, long-term stability and chronic functional performance of SPIFEC. Enhancing spatial selectivity in such devices could improve the efficacy and safety of bioelectronic systems, supporting applications like refined sensory feedback and motor control in neuroprosthetics.

## Supplementary Information

Supplementary Material 1

## Data Availability

Data sets generated during the current study are available from the corresponding author on reasonable request.

## Abbreviations

SPIFEC: Single penetrating interfascicular electrode (SPIF) added to an extraneural cuff (EC)
FBR: Foreign body response
PNI: Peripheral nerve interface
PEDOT:PSS: Poly(3,4-ethylenedioxythiophene):polystyrene sulfonate
CE: Conducting elastomer
PDMS: Polydimethylsiloxane
EIS: Electrochemical impedance spectroscopy
CV: Cyclic voltammetry
CIL: Charge-injection limit
TN: Tibial nerve
PN: Peroneal nerve
SN: Sural nerve
CT: Computed tomography
CNAP: Compound nerve action potentials
PE: Penetrating electrode
ESA: Electrical surface area
GSA: Geometrical surface area
