# Supplementary material for: Using a single penetrating interfascicular electrode to improve spatial selectivity of an extraneural polymeric cuff array
Source: Bioelectron Med. 2025 Dec 10;11:29. doi: 10.1186/s42234-025-00193-6 (PMC12690793; doi:10.1186/s42234-025-00193-6)

# **Supplementary**

Table S1 - Stimulation protocol parameters

| **Stimulation protocol** | **Type** | **Symmetry** | **Pulse width variation** | **Polarity (first phase at penetrating electrode)** | **Working electrode** | **Return electrode** |
| --- | --- | --- | --- | --- | --- | --- |
| **1** | Bipolar | Symmetric | Injection + return | Cathodic-first | Penetrating electrode | Extraneural electrode(s) |
| **2** | Bipolar | Asymmetric | Return | Cathodic-first | Penetrating electrode | Extraneural electrode(s) |
| **3** | Tripolar (2 sink + 1 source) | Asymmetric | Return | Cathodic-first | Penetrating electrode | Two extraneural electrodes |
| **4** | Tripolar (2 sources + 1 sink) | Asymmetric | Return | **Anodic-first** | Two extraneural electrodes | Penetrating electrode |
| **5** | Bipolar | Asymmetric | Injection | Cathodic-first | Penetrating electrode | Extraneural electrode(s) |

Five stimulation configurations were tested using both the penetrating and non-penetrating nerve cuffs.

In Table S1, the “Symmetry” column indicates whether the cathodic and anodic pulse widths were equal (symmetric) or different (asymmetric). This relates directly to the “Pulse width variation” column, which specifies whether the change in pulse width was applied to the injection (working) phase, the return phase, or both.

The “Polarity” column refers to the direction of the first phase at the penetrating electrode. For cathodic-first configurations, the penetrating electrode delivered the initial negative phase (acting as the current source), with the extraneural electrodes serving as returns. Conversely, for anodic-first configurations, the penetrating electrode received current during the first phase (acting as the sink), while the extraneural electrodes functioned as sources.

Table S2 - Three-way ANOVA table


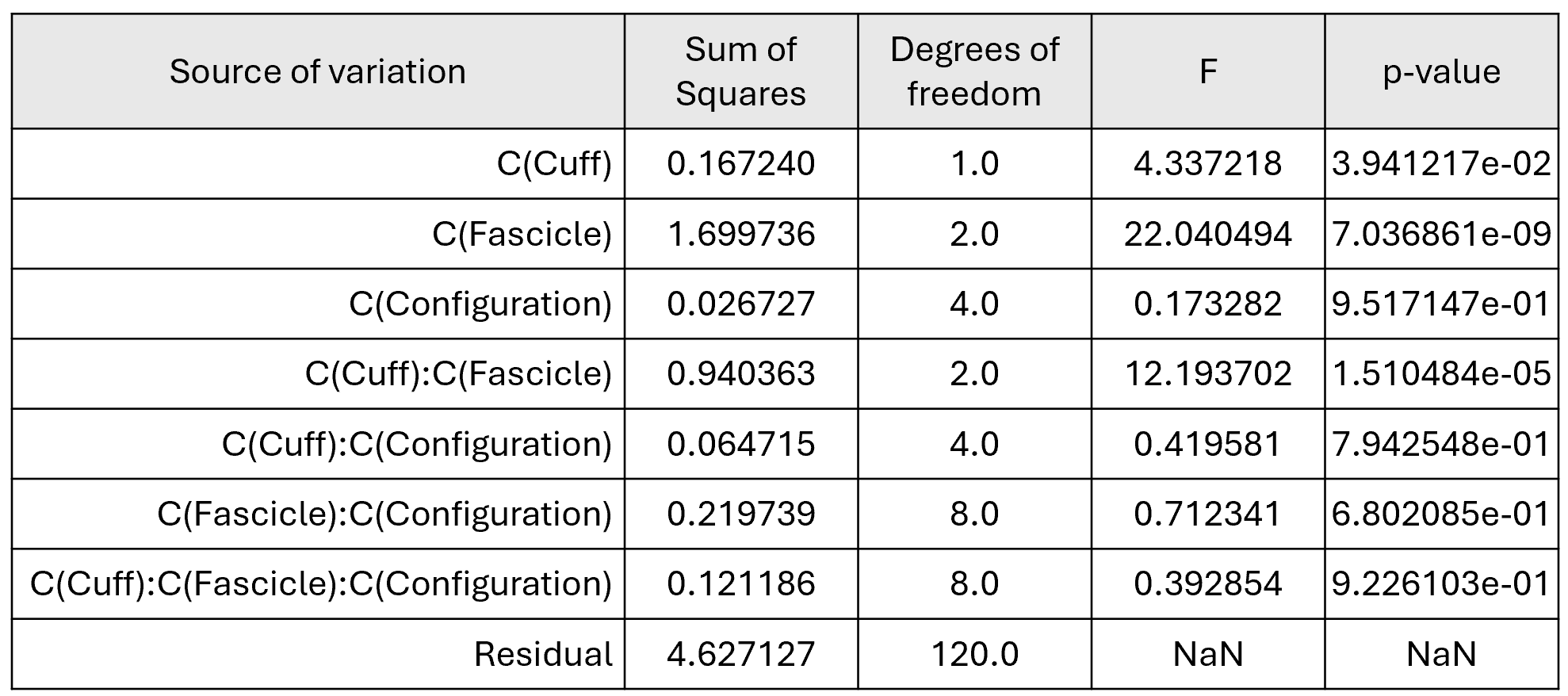


Table S3 – Post-hoc Tukey test on fascicle effect


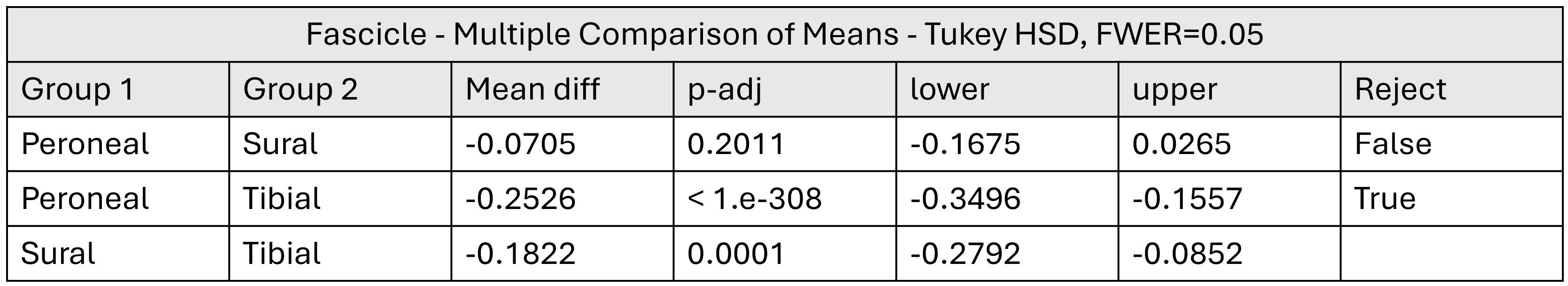


Table S4 - Post-hoc Tukey test on cuff effect


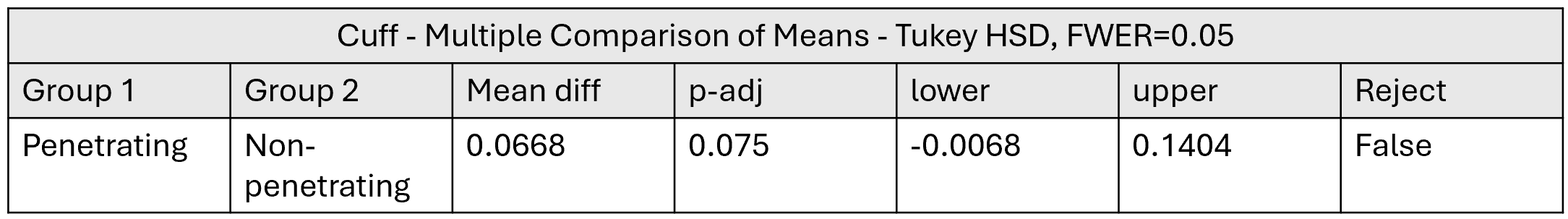


Table S5 - Post-hoc Tukey test on cuff-fascicle interaction. Note that P refers to the penetrating cuff and NP to the non-penetrating cuff


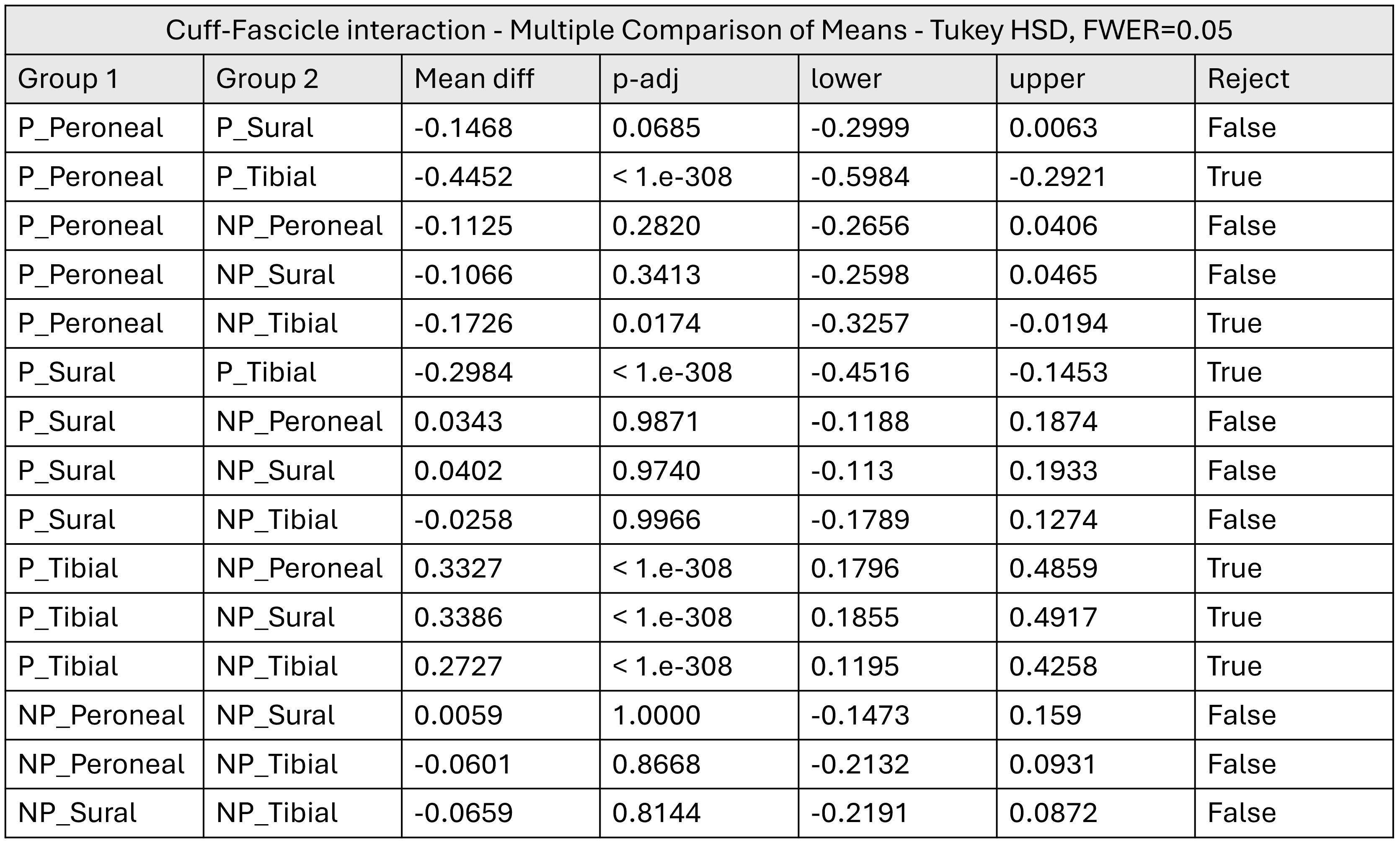

Supplement: Supplementary file 1 — Supplementary Material 1 [file 42234_2025_193_MOESM1_ESM.docx]
